# A new method for modeling coalescent processes with recombination

**DOI:** 10.1186/1471-2105-15-273

**Published:** 2014-08-11

**Authors:** Ying Wang, Ying Zhou, Linfeng Li, Xian Chen, Yuting Liu, Zhi-Ming Ma, Shuhua Xu

**Affiliations:** Institute of Applied Mathematics, Academy of Mathematics and Systems Science, Chinese Academy of Sciences, Beijing, 100190 China; Max Planck Independent Research Group on Population Genomics, Chinese Academy of Sciences and Max Planck Society (CAS-MPG) Partner Institute for Computational Biology, Shanghai Institutes for Biological Sciences, Chinese Academy of Sciences, Shanghai, 200031 China; School of Computer and Information Technology, Beijing Jiaotong University, Beijing, 100044 China; Department of Mathematics, School of Science, Beijing Jiaotong University, Beijing, 100044 China

**Keywords:** Recombination, Coalescence, Ancestral recombination graph (ARG), Sequentially markov coalescent (SMC)

## Abstract

**Background:**

Recombination plays an important role in the maintenance of genetic diversity in many types of organisms, especially diploid eukaryotes. Recombination can be studied and used to map diseases. However, recombination adds a great deal of complexity to the genetic information. This renders estimation of evolutionary parameters more difficult. After the coalescent process was formulated, models capable of describing recombination using graphs, such as ancestral recombination graphs (ARG) were also developed. There are two typical models based on which to simulate ARG: back-in-time model such as *ms* and spatial model including Wiuf&Hein’s, SMC, SMC’, and MaCS.

**Results:**

In this study, a new method of modeling coalescence with recombination, Spatial Coalescent simulator (SC), was developed, which considerably improved the algorithm described by Wiuf and Hein. The present algorithm constructs ARG spatially along the sequence, but it does not produce any redundant branches which are inevitable in Wiuf and Hein’s algorithm. Interestingly, the distribution of ARG generated by the present new algorithm is identical to that generated by a typical back-in-time model adopted by *ms*, an algorithm commonly used to model coalescence. It is here demonstrated that the existing approximate methods such as the sequentially Markov coalescent (SMC), a related method called SMC′, and Markovian coalescent simulator (MaCS) can be viewed as special cases of the present method. Using simulation analysis, the time to the most common ancestor (TMRCA) in the local trees of ARGs generated by the present algorithm was found to be closer to that produced by *ms* than time produced by MaCS. Sample-consistent ARGs can be generated using the present method. This may significantly reduce the computational burden.

**Conclusion:**

In summary, the present method and algorithm may facilitate the estimation and description of recombination in population genomics and evolutionary biology.

**Electronic supplementary material:**

The online version of this article (doi:10.1186/1471-2105-15-273) contains supplementary material, which is available to authorized users.

## Background

The genealogical relationship between sequences in a population is an important issue in recent analyses of the dynamics of sequence evolution at the population level. The genealogical relationship among a number of sampled sequences drawn from a particular generation of a large haploid population can be modeled using the Kingman’s coalescent process [1, 2]. This method has been successfully applied in haploid type data such as bacteria simulation, the estimation of population genetics parameters, and the inference of demographic events. However, the coalescent process involves no recombination and this cannot be ignored when studying diploid populations. For example, the histories of different loci in a genomic region may differ due to recombination events.

The first model of coalescence with recombination was described by Hudson [3]. This was shortly after Kingman’s coalescent process was formulated. Due to the increased complexity added by recombination, a graph rather than a single tree is needed to describe the genealogical relationship. This graph, called an ancestral recombination graph (ARG), is made up of many local coalescent trees [4]. An ARG can be considered a random graph. Each branch in the ARG represents a lineage that carries some ancestral material to the sample. Here, the term "ancestral material" refers to chromosomal regions that are eventually inherited by any of the samples of interest drawn from the present-day population. The node in ARG at which two branches converge denotes a coalescent event, and the node at which one branch splits into two denotes a recombination event.

An algorithm that can rapidly generate independent ARGs from populations evolving with both coalescence and recombination can be of great use. First, they can facilitate data analysis. Samples produced using various models can be combined with data to test hypotheses. Second, it can be used to estimate the recombination rate. The question of whether recombination events are clustered in hotspots is of enormous interest at present. It also has considerable relevance to the efficient design of association studies [5].

There are two main representative algorithms that can simulate ARG according to a given recombination rate. One is Hudson’s *ms* [6]. It is the simplest and is used in many applications. The other is Wiuf and Hein’s spatial algorithm [7]. These two algorithms stress different aspects of the process. The algorithm of *ms* has a Markovian structure and is computationally straightforward. Ancestral lineages related to the sampled chromosomes remain unchanged until coalescence or recombination. In contrast, Wiuf and Hein’s spatial approach of simulating genealogies along a sequence has a complex, non-Markovian structure. The distribution of the next local tree depends on all previous local trees rather than on the current genealogy alone. It begins with a coalescent tree at the left end of the sequence and adds more different local trees gradually along the sequence, which form part of the ARG. The algorithm terminates at the right end of the sequence when the full ARG is determined.

**Figure 1:**
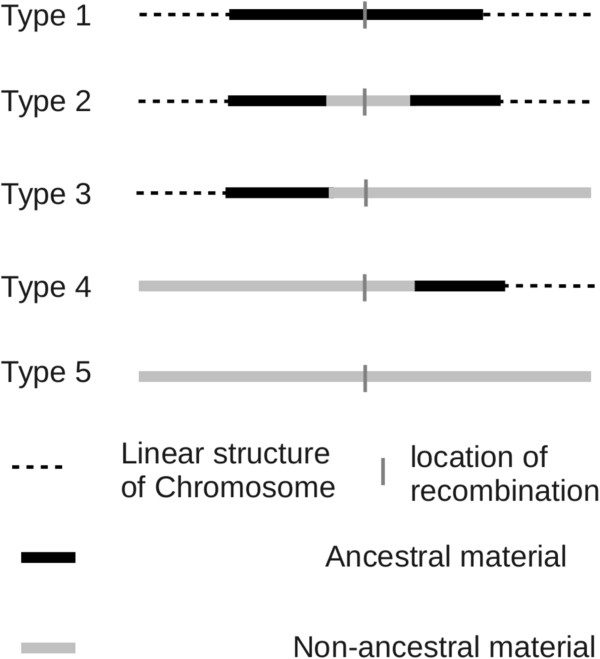
**Five types of recombination in the history of a population.**

To compare existing algorithms, the recombination events in history were classified into five types [8]: Type 1: recombination with breakpoint located in ancestral material; type 2: recombination with breakpoint located in non-ancestral material with ancestral material on both sides; type 3: recombination with breakpoint located in non-ancestral material with ancestral material on only the left side; type 4: recombination with breakpoint located in non-ancestral material with ancestral material on only the right side; type 5: recombination in an individual carrying no ancestral material. These five types of recombination are shown in Figure 1.

Because only type 1 and type 2 contribute to the gene structure of the sample, ARG should in principle contain only these two types of recombination and the branches containing other types of recombination are regarded as redundant ones in simulated ARG. It seems that *ms* is the briefest way to simulate the ARG according to its distribution because it considers only type 1 and type 2 recombination. Wiuf and Hein’s method also simulates the other three types of recombination, which may produce some redundant branches and increase computation burden in generating ARG. When simulating hundreds of thousands of ARGs with a large recombination rate is required (e.g. to estimate recombination rate of a long DNA sequence based on full likelihood), even *ms* is not efficient enough, neither is it easy to approximate. Although the original spatial algorithm of Wiuf and Hein’s method produces a lot of redundant branches, several approximate spatial algorithms have been developed to reduce the redundant branches in ARG and simulate large samples of long sequences [8–10].

Likelihood-based inference is one statistical method that is commonly used to apply the corresponding algorithm to estimations of the recombination rate. The likelihood can be estimated by simulating ARGs from the coalescent distribution given the recombination rate *r* and mutation rate *θ*. The simulated data can be examined to see if they match the observed data. By repeating this process many times with different values of *θ* and *r*, maximum likelihood estimations of the statistics can be obtained [9]. However, because the vast majority of ARGs is not consistent with the sample and contributes nothing to the likelihood, this naïve method is infeasible. With a complete history, it is easy to calculate both the probability of the data and of the history given the coalescent model and associated parameters. The central difficulty is that, from an essentially infinite set of histories that could give rise to the data, it is hard to find histories that are highly probable under the assumed model. There are two approaches that have been developed to handle this difficulty [11].

The first approach involves sophisticated Monte Carlo methods such as importance sampling [12] and Markov Chain Monte Carlo [5, 13, 14]. MCMC starts from an initial guess and then tends to make subsequent modifications that are more likely to be accepted, with a probability that is proportional to how likely they occur under the assumed model. Importance sampling approximates the optimal proposal density to calculate the likelihood. Both methods create bias towards the simulation of ARGs, which makes significant contributions to the likelihood.

As a supplementary method, the second approach of estimating recombination rate is to simplify or approximate the coalescent model itself. Based on Wiuf and Hein’s spatial point of view, McVean and Cardin developed sequentially Markov coalescent (SMC), an approximation of the standard coalescent process [9]. This algorithm reduces the topology simulated to a tree rather than a graph. If two ancestral lineages have no interval in common where they share ancestral material, they are not allowed to coalesce. By restricting coalescent events in this way, the resulting process has a Markovian structure in the sequential generation of genealogies along a chromosome. The SMC starts with a coalescent tree at the left-hand end of the sequence and progressively modifies the tree with recombination events as it moves to the right. Marjoram and Wall modified the approximation [8]. In their system, the old lineage is not removed until after the point of coalescence of the new one has been determined. This allows for the possibility that the new lineage coalesces with the one that was to be erased and that no change occurs in the genealogy. Chen, Marjoram, and Wall described an intermediate approach (MaCS), which is a compromise between the accuracy of the standard coalescence and the speed of the SMC [10]. In the SMC, coalescent events are restricted to edges within the last local tree only. While in MaCS, coalescent events are restricted to edges among any of the last *k* (denoted as the tree retention parameter) local trees. It models the relationships between recombination events that are physically close to each other and treats those that are far apart as independent.

The essence of these approximate methods is to simplify the recombination of type 2 events and the coalescence of lineages that contain distant ancestral material. These methods offer significant improvements with respect to computational efficiency and sequence length. However, the effects of these simplifications have not yet been clearly classified.

This paper reports the establishment of a new method of modeling coalescence with recombination. It offers several improvements over that of Wiuf and Hein’s method. A new algorithm based on the new model is proposed to generate ARGs equal to *ms*. Similar to the algorithm designed by Wiuf and Hein, the present algorithm constructs ARG spatially along the sequence [7]. However, it will not produce any redundant branches, which are inevitable in Wiuf and Hein’s algorithm. It is here suggested that the above approximate methods (SMC [9], SMC′ [8], MaCS [10]) be viewed as special cases of our new algorithm. Using simulated analysis, the present algorithm was compared to MaCS. The time to the most common ancestor (TMRCA) in the local trees of ARGs generated by the present algorithm was even more similar to that produced by *ms* than that produced by MaCS was. The present method can generate sample-consistent ARGs, which might significantly reduce the computational burden.

## Results

### Model assumptions

This present work was performed with the same assumptions as those made by Griffiths and Marjoram [15]:

1. A gene, here treated as a length of DNA, is represented by the unit interval [0,1).
2. The population is assumed to evolve through discrete generations in a Wright-Fisher manner, which means that each generation is of 2 *N* genes in size. As usual, time is measured in units of 2 *N* generations. *N* → ∞ and 4*Nr* → *ρ* remains fixed, where *r* is the regional recombination rate per generation per gene, and *ρ* is the global population recombination rate.
3. The present algorithm was designed under the infinite sites model, in which the mutation is independent of the coalescent with recombination so that it occurs with a Poisson distribution on the ARG.
4. In the present algorithm, a gene copies the genetic information of its parents if recombination does not occur. If recombination does occur, only one breakpoint is assumed and the genetic information of the gene comes from its parents. Specifically, each gene chooses its parents from the previous generation according to the following rules: a) with probability 1–*r*, a gene from the previous generation is uniformly chosen; b) with probability *r*, a recombination event occurs, and two genes are uniformly chosen from the previous generation; c) each gene chooses its parents independently.

Meanwhile, the position of the breakpoint *S* is selected (independent of the generating events of the other genes) according to a given distribution with density p(*s*). The intervals [0,*S*) and [*S,*1), which are from the first and second parents, respectively, form the offspring gene. Here the random variable *S* possesses a continuous distribution.

### Generation of ARG along sequences with *SC*

Generating ARG is key to simulating coalescent processes with recombination. Most studies of theoretical population genomics do not generate ARG, even though many algorithms have been developed to do so. These include Hudson’s *ms* [6], Wiuf and Hein’ algorithm [7], Chen et al.’s MaCS [10], SMC [9], and SMC′ [8]. These algorithms can be roughly classified into two categories according to the different methodologies they use. One generates ARG back in time. Hudson’s *ms* is a representative example [6]. It is the most accurate algorithm because of its complete ARG space. The other generates ARG by gradually constructing a series of local trees along the sequence, such as Wiuf and Hein’ algorithm [7], MaCS [10], SMC [9], and SMC′ [8]. These algorithms are collectively called spatial algorithms. Particularly, MaCS, SMC and SMC’ are approximate spatial algorithms, which can generate ARG with longer sequences than *ms* can because they lose some information during the generation of ARG space.

In this work, a new spatial algorithm called the Spatial Coalescence simulator (*SC*) is proposed. It generates ARG more accurately along the sequence than other spatial algorithms do. If *X*^*S*^ denotes [*0, S*] segments of the ARG, and denotes the local tree of S-site, then X^0^ = is a standard coalescent tree without recombination, and *X*^1^ is the total desired ARG. The basic idea underlying spatial algorithms is constructing the ARG from X^0^ to *X*^1^ step by step. This process can be generalized into the following brief steps and the full version can be found in Methods.

- Step 1. j = 0, *S*_0_ = 0, Build a standard coalescent tree *X*^0^.
- Step 2. Generate a breakpoint of recombination *S*_*j* + 1_ in the interval [*S*,1) and choose a location from the current ARG X^S^_*j*_ .
- Step 3. Build a new ARG *X*^S*j+*1^ by adding a new coalescent branch to the current ARG X^S*j*^, and the coalescent event begins from the selected location and ends to any position of X^S*j*^.
- Step 4. Repeat steps 2 to 4 until *S*_*j*+1_ > 1, and then take the total ARG *X*^1^ as the ARG *X*^S*j*^.

**Figure 2:**
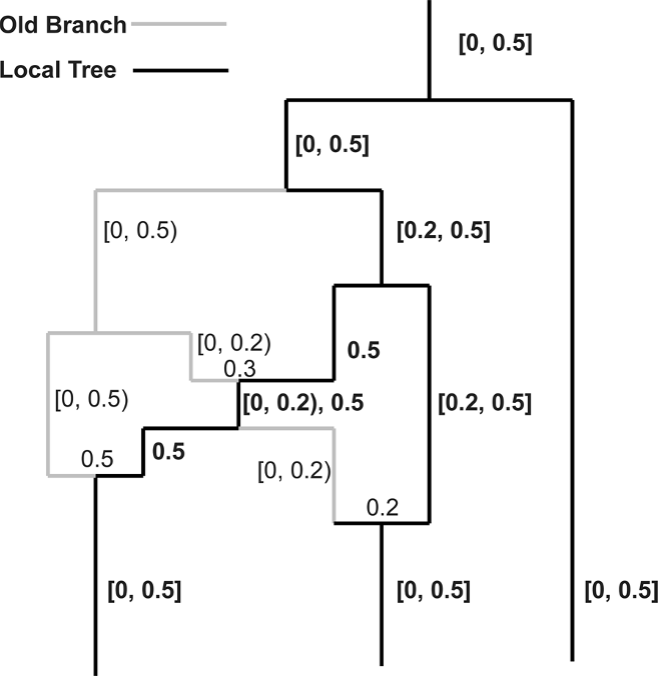
**An example of current ARG.** The graph displays the [0, 0.5] part of an ARG, the black lines represents branches constituting the current local tree, the gray lines represent all old branches, the numbers in brackets display intervals which denote the ancestral materials carried by nearby branches, the numbers without brackets denote recombination rates occur in the underlying nodes.

With a joint consideration of all the five classification types of recombination, we can reach the following conclusions: 1) the location of type 1 recombination must be on rather than the whole current ARG *X*^*Sj*^; 2) the location of type 2 recombination must be on the other branches of *X*^*Sj*^ which are called ‘old branches’ (see Figure 2 for an example of *X*^*Sj*^ and old branch); 3) not all recombination with location on old branches are type 2 recombination. Further, because each branch of contains ancestral material of *S*_*j*_-site, and the next break point is S_*j*+1_, therefore, each branch of contains ancestral material of [*S*_*j,*_*S*_*j*+1_), and it must be type 1 recombination. With respect to recombination on old branches, the only information that can be obtained is that there could be ancestral material on [0, *S*_*j*_) with an algorithm generating ARG without redundant branches when determining *X*^S*j+*1^. It is also certain that there must be no ancestral material on [*S*_*j,*_*S*_*j*+1_), but it is not clear whether there is ancestral material on [*S*_*j*+1_, 1).

Based on these conclusions described above, the following can be obtained:

1. Wiuf and Hein’s algorithm simulates type 1, 2, and other three types of recombination events because this algorithm fixes any recombination breakpoints without determining its type during step 2. It is therefore obvious that Wiuf and Hein’s algorithm generates more redundant information than *ms* (*ms* simulates types 1 and 2 but no other unnecessary recombination [8]).
2. MaCS simulates only type 1 recombination events and does not consider other types. This is because, during step 2, MaCS just fixes the type 1 recombination breakpoints by choosing a location from the current tree, but not the whole current ARG. In this way, MaCS ignores type 2 recombination information which *ms* does not.

Now the key problem for spatial algorithms is distinguishing type 2 from type 3 and adding useful recombination to the final total ARG.

To solve this type of problem, a new algorithm is here proposed to distinguish type 2 from type 3 events. In this algorithm, it is type 2 rather than type 3 recombination events that are entered into previous local trees after the latter type 1 breakpoints have been fixed. Specifically, the second step of this process differs from that of Wiuf&Hein’s, which we put recombination break points on the current local tree instead of the current ARG. This caused the type 1 recombination breakpoints to be fixed after step 2. Then step 3 is refined: A coalescent event beginning at the selected location was added to the current tree, and the end of the coalescent event is also located. If the location is on an old branch, then type 2 recombination breakpoints can be found back on this old branch. Finally, a full ARG can be built without missing any type 2 recombination events.

**Figure 3:**
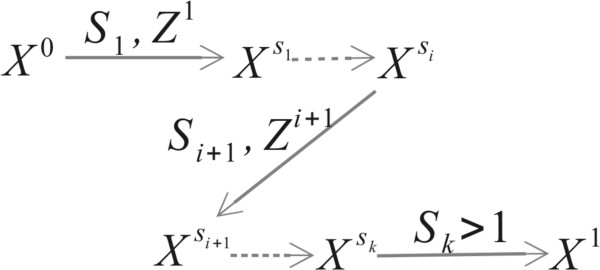
**Generation of ARG along sequence.** *X* ^*s*^ denotes [0, s] segments of the ARG, *S*_*i*_ denotes the *i* ^th^ type 1 recombination breakpoints and *Z* ^*i*^ denotes the branches added to *X* ^Si*-*1^. *X* ^Si^ is the collection of *Z* ^*i*^ and *X* ^Si*-*1^.

In this way, the new algorithm can be formulated as a sequence of random variables {(*S*_*i*_, *Z*^i^), i ≥ 0}, where *S*_*i*_ are the type 1 recombination breakpoints and *Z*^i^ describes the branches added at the break location of the ARG *X*^S*i-*1^ (Figure 3). They include type 2 recombination breakpoints and corresponding coalescent events.

In fact, the probability distribution on ARG space generated by this algorithm, which is based on a spatial model along a sequence, is identical to that produced by an algorithm based on the back-in-time model. To see this intuitively, we construct the whole ARG by constructing its constrain or projection on [0, s) from s = 0 to s = 1. The difference between the constrain of [0, *S*_*j*_) and [0, *S*_*j* + 1_) (*X*^S*j*^ and *X*^S*j+*1^, respectively) can be more than 1 branch. That’s because if there is a recombination with break point in [*S*_*j*_, *S*_*j* + 1_), we can obtain it at least when we obtained *X*^S*j+*1^. So, in order to get *X*^S*j+*1^, we choose to add one or more than one branches to *X*^S*j*^.

The experimental proof of the above identification can be seen in the performance of SC and SC-sample section. The details of the procedures associated with the present algorithm can be found in the methods section. The Mathematical framework of the algorithm is provided in another paper [16].

### Generate sample-consistent ARG

The idea of sample-consistent ARG first appeared in the work of Song [17]. In their study, ARG was used to estimate the minimal number of recombination events. ARG with the minimal recombination numbers definitely helped the study of recombination. We do not think that ARG with minimal number of recombination represents a true ARG. Therefore, we attempt to design an algorithm that can model a group of ARG which are consistent with sample in a reasonable way, rather than simply produce all the ARGs in the whole ARG space, neither does it generate ARGs with minimal number of recombination events. We believe that by this way our method generates sample-consistent ARGs which reflect true genealogical information of the samples and it will definitely help us estimate parameters of population demographic history.

In the present study, we further modified the *SC* algorithm to generate sample-consistent ARG. One ARG is called sample-consistent if the given sample of sequences can be generated using the ARG under the infinite sites model, which means that each site on the sequences can be explained using the local tree of the ARG. An example of sample-consistent ARG is given in Additional file 1: Figure S1. Notably, sample-consistent ARG is a very small part of the full ARG space. In our simulation study with *ms*, less than 10 sample-consistent ARGs were found in millions of simulated ARGs. The algorithm described above, *SC*, was modified slightly. We therefore developed a new algorithm called *SC*-sample, with which sample-consistent ARGs can be generated. Suppose there are sample sequences with 0 and 1 coded for different alleles, then, the procedure of this *SC*-sample algorithm can be described as follows:

- Step 1 Generate a standard coalescent tree *X*^*0*^, which is consistent with the left site of the sequence.
- Step 2 One by one, confirm whether the following sites are consistent with the current tree *X*^*0*^, and find the first inconsistent site *P*_1_. Then generate a breakpoint of type 1 recombination *S*_1_ in the interval [0, *P*_1_], and choose a location from the current ARG *X*^*0*^.
- Step 3 Build a new ARG X^S1^ by adding branches to *X*^*0*^ at the chosen location to make the local tree after *S*_*1*_ consistent with the first site after *S*_*1*_.

Repeat step 2 to 3 until *S*_*j* + 1_ > 1 to get an ARG consistent with the sample for the full sequence.

### Performance of *SC*and *SC*-sample

**Figure 4:**
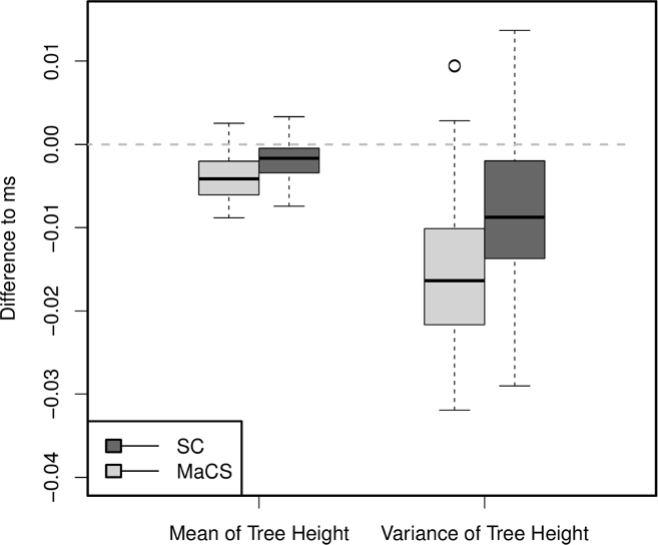
**Comparison of differences in the mean and variance of the first 100 local trees’ height between SC and Macs using** ***ms*** **as a control.** Boxplot with 75% quantile and 25% quantile as top border and the bottom border, respectively.

It is here proven that the distribution, i.e. the statistical properties, of the ARG generated by *SC* coincides with that generated by *ms* [6]. MaCS (*h* = *L*) was found to simulate all the type 1 but no type 2 recombination. In order to determine the equivalence of *SC* and *ms* and assess the influence of type 2 recombination, the mean and variance of the first 100 local trees generated by *SC*, *ms*, and MaCS were compared (Figure 4, Additional file 2: Figure S2). The difference between *SC* and *ms* and that between MaCS and *ms* were determined separately using the difference of mean of the local tree’s height and variance of the local tree’s height, respecting the mean and variance of *ms*’s local tree’s height, details in **Methods**. Twenty haplotypes were simulated for a total of 100,000 rounds with *ρ*(=4*N*_*e*_*Lr*_*p*_) of 100 at *L* = 167 kb.

The present results show that *SC* is more similar to *ms* with respect to both the mean and variance of the TMRCA than MaCS is. This confirms that the results of the theoretical study that ARGs generated by *SC* and *ms* share similar statistical properties, indicating that *SC* performs better than MaCS in the modeling of ARG.

In practice, it is very important to take into account the time cost and the RAM usage of the computer programs that implement the algorithm. The two values were the average of 10 replicates between *SC* and *ms*. Results are shown in Table 1 for *n* = 20, *n* = 100 and *n* = 1000 sequences using different recombination rate parameters *ρ* = 4*N*_*e*_*Lr*_*p*_. The results show that *ms* runs slightly faster but needs more RAM, though *SC* and ms share similar statistical properties. In addition, even the latest version of *ms* cannot simulate sequences as long as those *SC* does.

**Table 1:** **Comparison of average time cost and memory usage between** ***SC*** **and** ***ms***

Sample size	Region (***L***)	***ρ***(4***N***_***e***_ ***Lr***_***p***_)	***SC***	***ms***
20	10 Mb	1000	45 s (14 MB)	13 s (63 MB)
	10 Mb	10,000	3 h 43 min 22 s (210 MB)	2 h 3 min 53 s (344 MB)
	100 Mb	1000	57 s (14 MB)	N/A
	100 Mb	10,000	4 h 30 min 18 s (238 MB)	N/A
100	10 Mb	1000	1 min 57 s (19 MB)	23 s (148 MB)
	10 Mb	10,000	7 h 11 min 34 s (281 MB)	N/A
	100 Mb	1000	2 min 11 s (20 MB)	N/A
	100 Mb	10,000	7 h 13 min 3 s (289 MB)	N/A
1000	1 Mb	1000	3 min 13 s (26 MB)	12 s (215 MB)
	10 Mb		3 min 20 s (26 MB)	N/A

Memory usage (MB) is in parentheses. Mb: sequence length in million base pairs. N/A entries denote test cases that were terminated when we ran on a server with 4 CPUs of 2.40GHz and 24GB for total memory. ***ρ*** denotes the population recombination rate.

**Figure 5:**
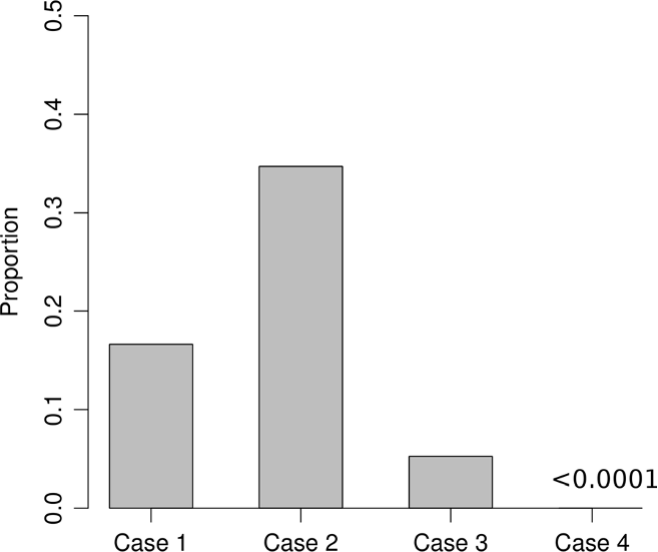
**Ratio of strictly sample-consistent ARGs to all ARGs.** ARGs are not considered strictly sample-consistent unless they are both sample-consistent and the number of type 1 recombination of that ARG is within 10% of estimate using 100,000 simulations in 4 different cases. Case 1: ρ = 10, μ = 10 with *SC*-sample. Case 2: ρ = 50, μ = 50 with *SC*-sample. Case 3: ρ = 16.9,μ = 7.5 with *SC*-sample, which employ the mutation rate and recombination rate in human. Case 4: ρ = 10, μ = 10 with *SC*.

Next, the performance of *SC*-sample was evaluated. Two hundred samples were generated with different recombination and mutation rates. Then *SC*-sample was used to generate 1000 ARGs that were consistent with each sample. Consistency was confirmed by adding mutations to the ARG. Then the recombination and mutation parameters of humans were used to repeat the experiment shown above. Then 100 independent genealogies of 20 chromosomes were generated and complete sequences were simulated, each with an interval of 30 kb. A constant *c* = 1.13 *cM*/*Mb* was assumed, which is the sex-averaged recombination rate [11]. The per-site mutation rate was assumed to be 1× 10^-8^, and the effective population size was assumed to be 12,500. The ratio of strictly sample-consistent ARG generated by SC-sample and SC were calculated, the results are shown in Figure 5.

**Figure 6:**
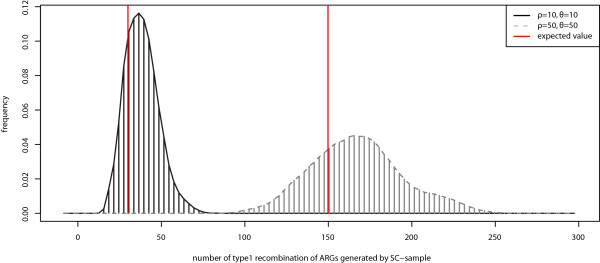
**Distribution of number of type1 recombination in ARGs generated with SC - sample.** The red vertical line indicates the expected number of type 1 recombination in a particular scenario**.**

Because there is no available sample-consistent algorithm for comparison, the performance of the *SC*-sample algorithm could not be fully evaluated. However, the result shows that sample-consistent ARGs randomly chosen reveal directly some recombination information of the sample. The numbers of recombination events of the sample-consistent ARGs are close to the mean number of samples (Figure 6). That means the *SC*-sample generated sufficient numbers of ARGs which are close to the true recombination events without generating many ARGs that were inconsistent with samples. *SC*-samples may be very helpful in estimating the recombination rate in the future, considering that full sequence data are now becoming available.

## Discussion

In the present study, a new method for modeling coalescent processes with recombination was developed. This method offers some improvements over Wiuf and Hein’s method. It covers all the commonly used spatial simulation algorithms with approximations, i.e. SMC, SMC′, and MaCS. Based on this method, a new algorithm, *SC*, was developed for the simulation of ARG and the generation of data. This algorithm has been shown to be able to simulate ARG with the same distribution as that produced by a back-in-time simulation algorithm. Another relevant algorithm, *SC*-sample, was also developed for generating sample-consistent ARG. The present method and algorithms have considerable potential to facilitate modeling and statistical inference of recombination.

Another study showed that the distribution of ARG generated by the new algorithm is identical to that generated by a typical back-in-time model [16]. In this study, computer simulation experiments confirmed that this feature was common to both the back-in-time method and the along-sequence method with respect to the simulation of ARG. In practice, *SC* takes slightly more time than *ms* for the same simulation but use less RAM. However, *ms* does not work as well as *SC* when the sequence is very long (e.g., 100 Mb). These comparisons indicate that neither of these two methods can generate ARG of long sample sequences in a satisfactory manner. Considering the above situation and rapid accumulation of huge genomic data, one possible solution or a tradeoff could be effected by approximating ARG to replace full ARG.

Several approximate methods have been developed for the simulation of ARG, such as the sequentially Markov coalescent (SMC), a related method called SMC′ and Markovian coalescent simulator (MaCS). These existing methods can be considered as special cases of the present method. Marjoram and Wall classified recombination into 5 types [8]. However, these methods all ignore type 2 recombination and some of the coalescent events associated with it. This may affect ARG reconstruction and statistical inference of recombination.

The influence of type 2 recombination on ARG was assessed by comparing the statistical properties of *ms*, *SC*, and MaCS (h = L). It was concluded that ignoring type 2 recombination would reduce both the mean and variance of the times to MRCA, although the reduction is not very remarkable. These results indicated that type 1 recombination might play a more important role in history than type 2 recombination. However, type 2 recombination was found to be present extensively and much more common than type 1 when long sequences are considered and simulated, suggesting that this type 2 recombination should not be ignored in simulation, especially for the simulation of long sequences. Therefore, an algorithm that takes into account type 2 recombination should be used for the simulation of long sequences. This is one of the reasons why the *SC* algorithm was developed.

During the simulation of recombination, regardless of which algorithm was used, the process is complex and time consuming. Many non-sample-consistent ARGs are generated. For this reason, a new concept, sample-consistent ARG, was developed, and an algorithm, *SC*-sample, was used to simulate sample-consistent ARG. However, on the one hand, simulations based on *SC*-sample save a considerable amount of time and significantly increase efficiency over that of any non-sample-consistent algorithm.

Taken together, the two algorithms developed in this study improved the modeling of coalescence with recombination. In a future study, new approximated methods should be developed to handle large-scale simulation of big data. Coalescence with recombination can be modeled using a random sequence {(*S*_*i*_, *Z*^*i*^) : *i* ≥ 0}. These different methods of approximation actually use different *S*_*i*_ and *Z*^*i*^. One possible solution is to approximate the random sequence {(*S*_*i*_, *Z*^*i*^) : *i* ≥ 0} from the mathematical aspects. These methods, when well established, can greatly facilitate studies of recombination modeling and recombination rate estimation.

## Conclusions

In this study, we developed a new method for modeling coalescent processes with recombination, and we demonstrated that our method has comparable performances with *ms* in generating ARG, a computer program commonly used to simulate coalescence. An outstanding feature of our method is that it does not produce any redundant branches which are inevitable in Wiuf and Hein’s algorithm. In addition, our method can generate sample-consistent ARGs. Interestingly, we elucidated that the existing approximate methods (SMC, SMC’, MaCS) are all special cases of our method. We believe our new method and algorithm will facilitate the modeling of recombination and advance our understanding of evolution of recombination events within and between populations.

## Methods

### SC

**Figure 7:**
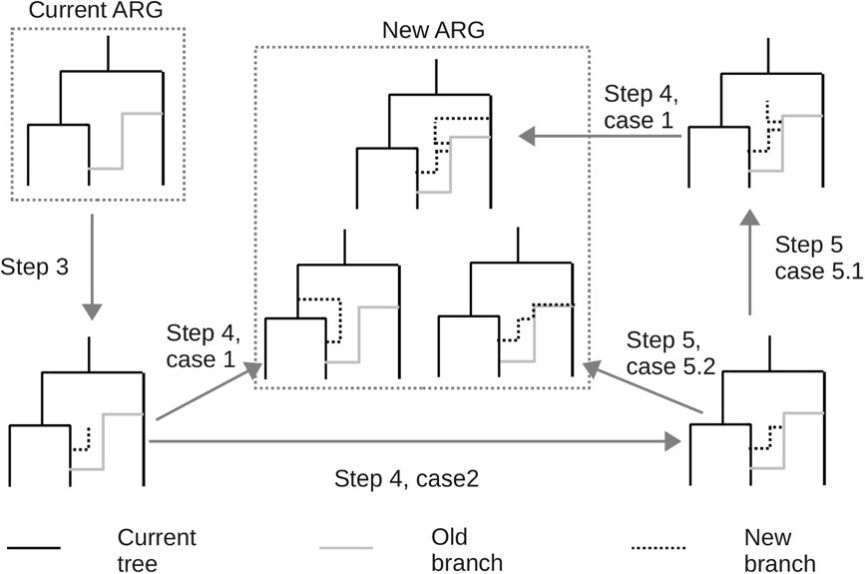
**Updated steps of** ***SC*** **to generate new ARG from current ARG.** The same step numbers and case numbers as in the methods section are used here. Step 3 is a new type 1 recombination created on the current ARG. In step 4, the new branch coalesces into an old branch or a branch on current tree. If the new branch coalesces into the current tree, a new ARG has been constructed. If the new branch coalesces into an old branch, then there are two cases, case 5.1 and case 5.2. In case 5.2, a new ARG is generated. In case 5.1, a new branch is generated which could be dealt with in step 4. When a new ARG is generated, it turns into current ARG and a new round begins. For more details, see the Methods section.

As a modified version of MaCS, *SC* can be outlined as follows. The algorithm can recursively construct part graph X^*Si*^ and each branch can be assigned some label *k* ≤ *i*. All the branches with label *i* form the local tree . This procedure is explained in Figure 7. Throughout the paper, we use s to denote a site on DNA sequence and t to denote the time that corresponds to certain locations on the ARG. In the following steps, step 1 is initializing, step 2–6 use a big circulation to construct the full ARG. Step 2 is to find the type 1 recombination break points and define the end condition of the big circulation, step 3 is to choose the location on the current tree of the type 1 recombination, step 4 is to consider the coalescence of the new branch caused by the type 1 recombination, step 5 is to use a small circulation to find all the type 2 recombination, and step 6 is to update the label for differing current tree and the old branches and goes to another bid circulation. Additional file 3: Figure S3 shows an example of the SC method.

Step 1. Construct a standard coalescent tree (c.f. [2]) at the position *S*_0_ = 0 (the left endpoint of the sequence) and assign each branch of the tree with label 0. Let .

Step 2. Assume that  has already been constructed along with local tree . Take the next recombination point *S*_*i* +__1_ along the sequence according to the distribution


Here,  is the total branch length of the current local tree , *ρ* is global population recombination rate, and p(*u*) is the density of the distribution of break point (see Model Assumptions for the explanation of p(*u*)). If *S*_*i* + 1_ ≥ 1, break; otherwise, go to step 3.

Step 3. Uniformly choose a recombination location on . Let *j* = 0, and let  denote the latitude (i.e. the height from the bottom to the location) of the chosen location.

Step 4. At the site of recombination, a new branch with label *i* + 1 is created by forking off the recombination node and moving backward in time (i.e. along the direction of increasing latitude). With equal exponential rate 1, the new branch will tend to coalesce to each branch in  that has a higher latitude than . In this way, if there are *l* branches in  at the current latitude, then the time before coalescence is exponentially distributed with parameter *l*. Note that at different latitudes there may be a different number of *l* of branches. Let the branch to which the new branch coalesces be called EDGE, and let  be the latitude of the coalescent point and *j* = *j* + 1.

Step 5. If the EDGE is labeled with *i*, which means the EDGE has coalesced to the current tree, go to step 6; if the EDGE is labeled with a *k* and *k* is less than *i*, which means the EDGE has coalesced to an old lineage, then a potential recombination event should be considered. The waiting time *t* of the possible recombination event on the EDGE is exponentially distributed with parameter .

Case 5.1. If  is less than the latitude of the upper node of the EDGE which is denoted by *H*, then it is the next recombination location. Let . The part of the branch above  is no longer called EDGE. Let *j* = *j* + 1 and go to step 4.

Case 5.2. If , choose the upper edge of the current EDGE with larger labels to be the next EDGE. Let , *j* = *j* + 1 and go to step 5.

Step 6. Let  be the collection of all the branches in  and all the new branches labeled *i* +1. Starting from each node 1 ≤ *m* ≤ *n* at the bottom of the graph, specify a path moving along the edges in  increasing in latitude until it reaches the top of the graph. Whenever a recombination node is encountered, choose the edge with the larger label. The collection of all the paths then forms the local tree . Update all the branches in  with label *i* + 1.

It is here noted that step 5 shows the key differences between the present method and other spatial algorithms. This step finds the missing type 2 recombination events. Essentially, the present algorithm gives the new branch a chance to leave when it coalesces into an old branch.

**Figure 8:**
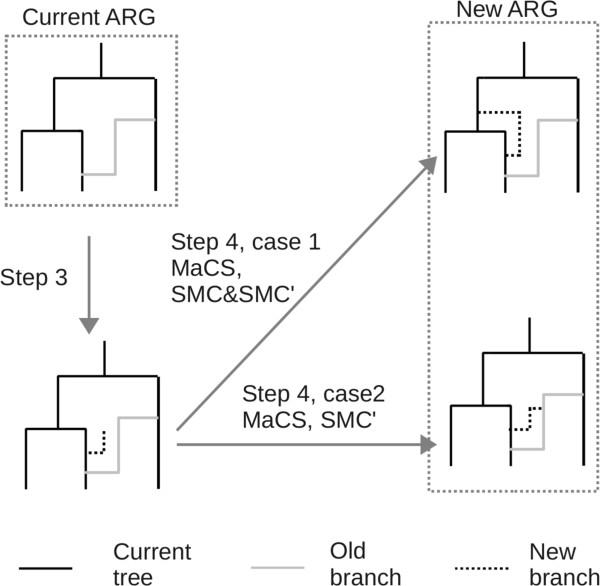
**A schematic diagram of the update steps of SMC, SMC′, and MaCS under our framework.** Regardless of which branch the new branch coalesces into in Step 4, a new ARG is constructed.

Actually, the existing approximating algorithms SMC, SMC′, MaCS can all be considered special cases of the present random sequence framework. The only difference is that these algorithms use a simpler *Z*^*i*^ than the present method does. These values are approximated as *Z*^*i*^ - *SMC*, *Z*^*i*^ - *SMC*', and *Z*^*i*^ - *MaCS*, respectively. One of the main differences lies in the fact that *Z*^*i*^ may construct many branches while *Z*^*i*^ - *SMC*, *Z*^*i*^ - *SMC*', and *Z*^*i*^ - *MaCS* each constructs only one branch (Figure 8). When a new branch coalesces to a branch with label of whose value is less than *i*, *SC* allows the branch to leave  due to the missing type 2 recombination, while the other three algorithms ignore these missing recombination and do not allow the branch to leave . The other difference is that, in step 4 (see above), in *SC*, the new branch can coalesce to each branch in  and the other three algorithms only allow the new branch to coalesce to certain branches in . SMC allows the new branch to coalesce to the branches with label *i* except for the branch where the recombination occurs, while SMC′ allows the new branch to coalesce to the branches with label *i* and MaCS restricts the coalesced branch to those with labels larger than *i* – *k*, where *k* is a fixed integer. In summary, all these existing approximation algorithms used a simpler version of *Z*^*i*^ than the present algorithm, and *Z*^*i*^ only allows the new branch to coalesce once to some particular branches.

### *SC*-sample

In order to relate a coalescent tree to the sample, we assign a value of the sample to each leaf node. Suppose the sample sequences are coded by 0/1, we can value the other nodes of the tree by 0 or 1. There should be a mutation existing on an edge if the values of the top and bottom nodes of it are different. The mutation edges are different with different value schemes. So there must be some schemes which make the coalescent tree with the minimum mutation number (MMN). The following is an algorithm to get the MMN.

- Step 1. Value the coalescent tree from bottom to the top according to the following rules: (a) Value a node with 1 if its two son nodes are all with value 1. (b) Value a node with 0 if its two son nodes are all with value 0. (d) Value a node with 2 if one of its two son nodes is with value 0, the other is 1. (e) Value a node with 1 if one of its two son nodes is with value 1, the other is 2. (f) Value a node with 0 if one of its two son nodes is with value 0, the other is 2. (g) Value a node with 2 if its two son nodes both have value 2.
- Step 2. For the top node of the coalescent tree, (a) change its value to 0 if it is valued with 2, (b) remain its value if it is valued with 0 or 1.
- Step 3. Revalue each 2-valued node with its parent node’s value.
- Step 4. The number of the mutation edges is the MMN.

See Additional file 4: Figure S4 for an example of the MMN algorithm.

Based on *SC*, a method capable of directly generating ARG which is consistent with the sample sequences is implemented. The basic idea is to pose some constraints during the gradual constructing of ARG to make sure that every local tree of the ARG is sample-consistent. The algorithm *SC*-sample is a modified version of *SC*. The differences between *SC*-sample and *SC* are outlined as follows:

- Step 1. At position *S*_0_ = 0, a coalescent tree *T*_0_ is constructed as a modification of the standard coalescent: (a) Give the value of the left site (first site) of the sample to the leaf node. (b) Randomly choose two nodes with the same value to coalesce and give the same value to the parent node. (c) When only one node with value 0 or 1 remains, the simulation becomes the standard coalescent simulation.
- Step 2. Assign each leaf node of the current tree a value of the sites after the current position of the sample, and then use the above MMN algorithm to assign every node of the current tree by making sure the number of edges with the value of the top and bottom nodes difference is minimal. In this way, each node on the current tree is valued by a 0–1 vector. Edges that have different values at the top and bottom nodes at a given are called mutation edges of the site.
- Step 3. Denote the first site that has more than one mutation edges as *P*_*i* + 1_. The next recombination point *S*_*i* + 1_ is uniformly chosen between *S*_*i*_ and *P*_*i* + 1_ (or *S*_*i* + 1_ is regenerated as before until *S*_*i* + 1_ < *P*_*i* + 1_).
- Step 4. Randomly choose a recombination site on the mutation edges of the site at position *P*_*i* + 1_.
- Step 5. The new lineage can only coalesce into 3 different types of edges: type A, the edges in the current tree with their values from *S*_*i*_ to *P*_*i* + 1_ are the same, type B, an old branch that leads to type A edges in the current tree, type C, a branch beyond the local MRCA.

**Figure 9:**
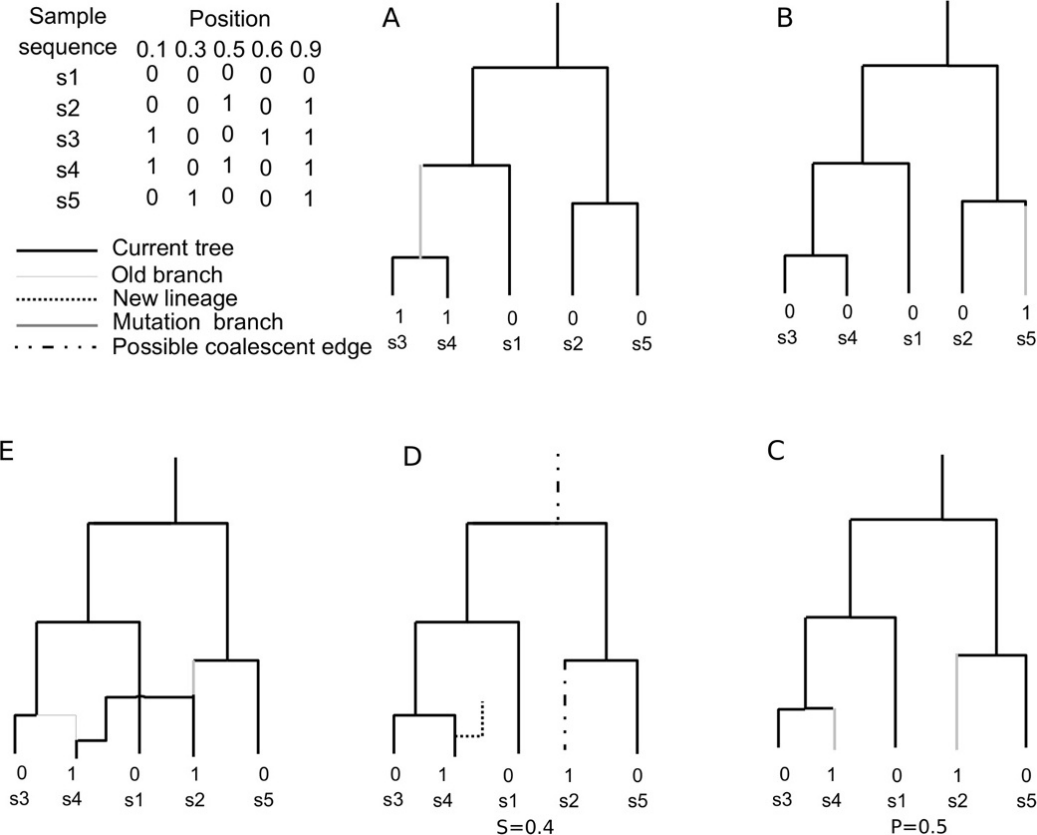
**Generation of sample-consistent ARG with SC-sample. A)** Generation of a binary tree is consistent with the left site. **B)** Determine whether the current local tree is consistent with the second site, the answer is yes since there is only one mutant edge. **C)** Determine whether the current local tree is consistent with the third site. This tree is not consistent because there are two mutant edges, so P_1_ = 0.5. **D)** Generate the next recombination point that is uniform on [0, P_1_] and obtain P_2_ = 0.4. The dotted lines are the branches onto which the new branches are supposed to coalesce. **E)** The new branch coalesces and the [0, 0.4] part of the ARG is simulated.

The other parts between the *SC* and *SC*-sample methods are the same. Figure 9 shows the *SC*-sample dynamically.

Uniform distribution was used when finding the next recombination point because the algorithm was designed to be independent of a prior recombination rate. Any ARG generated using this method can be viewed as a randomly selected ARG consistent with the sample. In principle, all kinds of algorithms can be modified. These can be considered special cases in our model (such as SMC and MaCS) to generate ARG consistent with the sample.

### Calculation of the differences among ARGs by the mean and variance of the heights of local trees

In order to study the differences among the ARGs generated using *SC*, MaCS, and *ms*, the mean and variance of the heights of the first 100 local trees were compared. Considering that the mean and variance of the tree height vary from the first to the last local tree, a new method is proposed to measure the difference of ARGs. With the mean values of the *i*^th^ local tree’s height generated by *SC* and *ms*, the difference between *SC* and *ms* was calculated as follows:


For the variance of the *i*^th^ local tree’s height, the difference is as follows:


Here,  represents mean height of the *i*^th^ local tree generated by *SC*. It  represents variance of the height of the *i*^th^ local tree. So, based on the mean and variance of the local tree’s height, the difference in ARGs generated by difference algorithm can be estimated.

### Availability and requirements

The algorithm for *SC* and *SC*-sample are implemented in C++ by modifying the code of MaCS. *SC* is implemented by the same set of demographic models used in *ms*, but *SC*-sample can only handle the classical homogeneous effective population size model (constant population size). Like MaCS, *SC* considers variations in recombination rate and intragenic gene conversion using a piecewise constant model and intragenic gene conversion, respectively [18]. The source code of SC and SC-sample can be downloaded from the website: http://www.picb.ac.cn/PGG/resource.php

-  Project name: SC and SC-Sample
-  Project home page: http://www.picb.ac.cn/PGG/resource.php
-  Operating system: GNU/Linux
-  Programming language: C++
-  Other requirements: g++ version 4.4.6 or higher; boost version 1.41 or higher
-  License: GNU GPL
-  Any restrictions to use by non-academics: license needed

## Electronic supplementary material

Additional file 1: Figure S1: An example of a sample-consistent local tree. Each leaf denotes one site of a gene which is coded by 0/1. A sample-consistent local tree denotes a binary tree that follows the infinite-site model, in which all the nodes labeled 1 coalesce first or all the nodes labeled by 0 coalesce first. (PDF 17 KB)

Additional file 2: Figure S2: Comparison of differences in the mean and variance of the first 100 local trees’ height between SC and Macs using *ms* as a control. Boxplot with 75% quantile and 25% quantile as top border and the bottom border, respectively. Twenty haplotypes were simulated for a total of 10,000 rounds with *ρ*(=4*N*_*e*_ *Lr*_*p*_) of 1000 at *L* = 167 kb. (PDF 5 KB)

Additional file 3: Figure S3: An example of SC method. An example of ARG. B,C and D describe the way of generating the ARG. The black thick branches make up the current tree. The gray branches are all old branches. The dashed lines are the path of the new branch. The black thin are un-simulated branches. The numbers in brackets display intervals which denote the ancestral materials carried by nearby branches. The numbers without brackets denote the recombination rates occur in the underlying nodes. In B,C, D, the numbers near edges are the labels of the SC method. (PDF 190 KB)

Additional file 4: Figure S4: An example of the MMN algorithm. Step 1, value the leaf nodes with the sample. Step 2, value each node from bottom to top. Step 3, revalue each 2-valued node. Step 4, thick lines denotes mutation branches, get the MMN = 3. (PDF 54 KB)
