# Supplementary figures and images for: A new method for modeling coalescent processes with recombination
Source: BMC Bioinformatics. 2014 Aug 11;15(1):273. doi: 10.1186/1471-2105-15-273 (PMC4137079; doi:10.1186/1471-2105-15-273)

Mutation

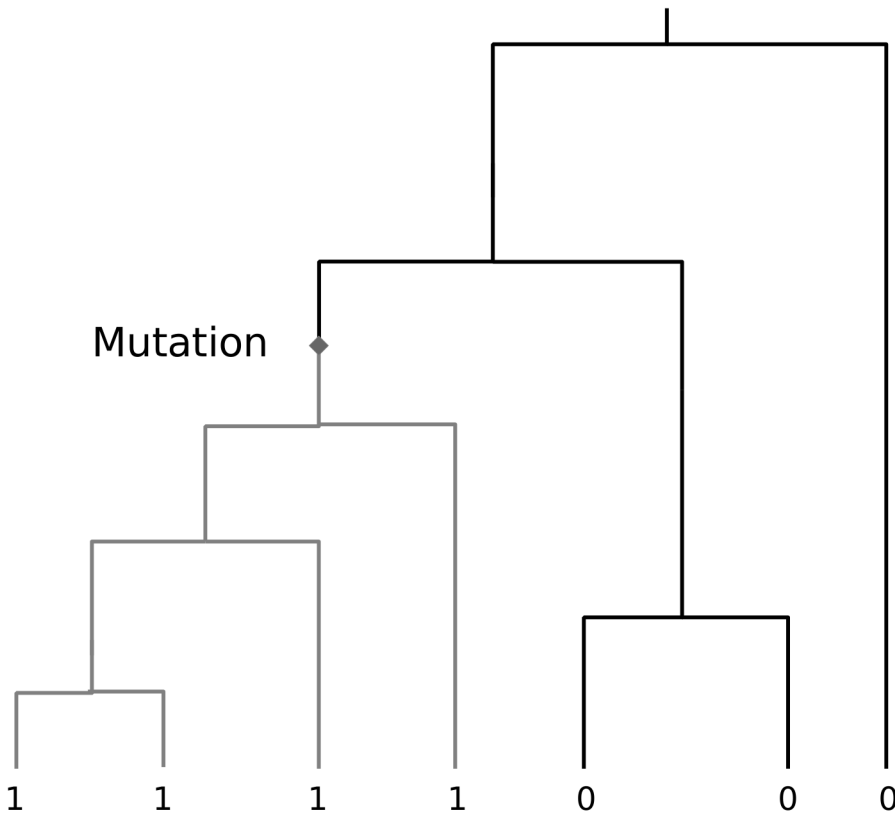

Supplement: Supplementary file 1 — Additional file 1: Figure S1: An example of a sample-consistent local tree. Each leaf denotes one site of a gene which is coded by 0/1. A sample-consistent local tree denotes a binary tree that follows the infinite-site model, in which all the nodes labeled 1 coalesce first or all the nodes labeled by 0 coalesce first. (PDF 17 KB) [file 12859_2014_6539_MOESM1_ESM.pdf]

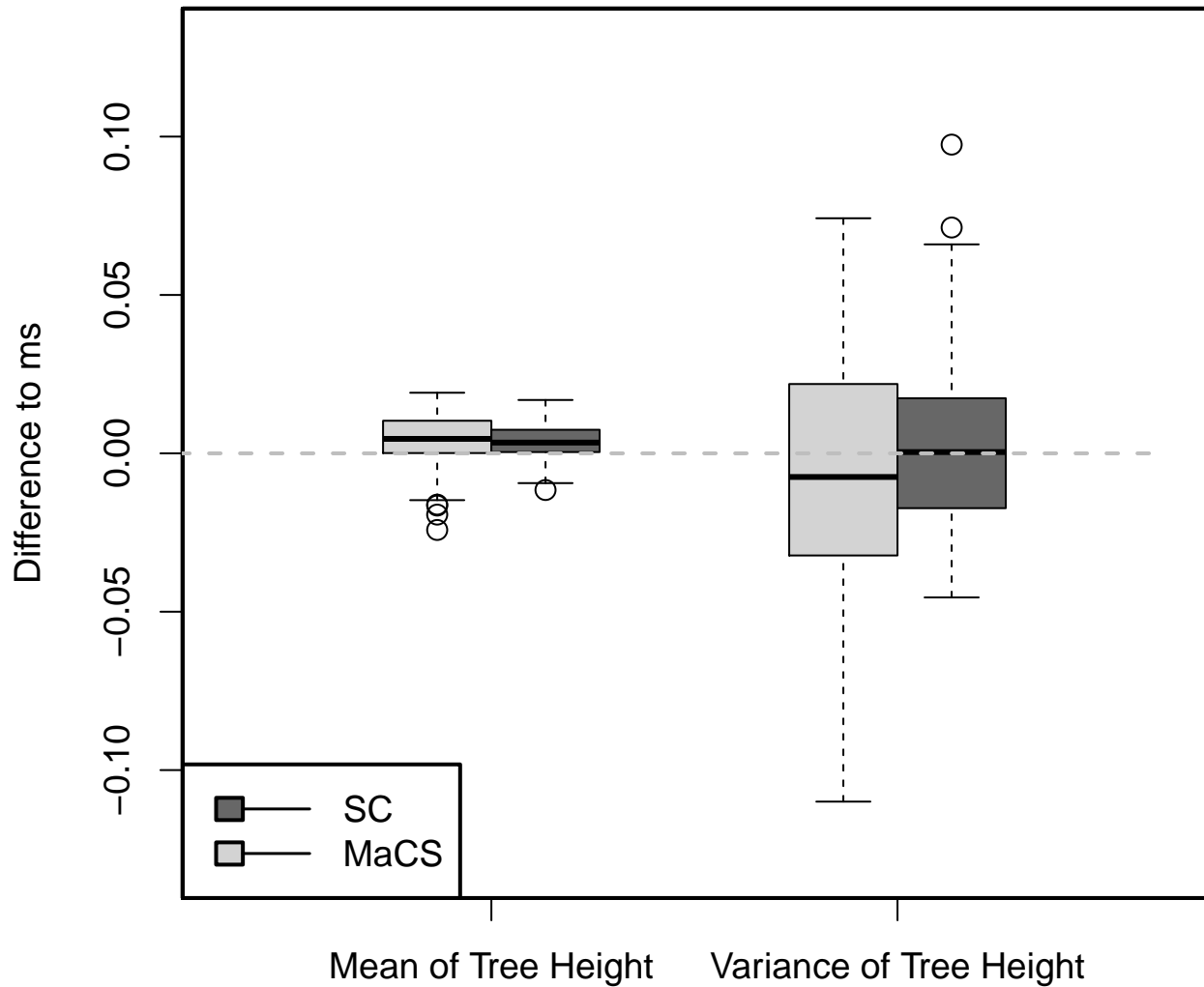

Supplement: Supplementary file 2 — Additional file 2: Figure S2: Comparison of differences in the mean and variance of the first 100 local trees’ height between SC and Macs using ms as a control. Boxplot with 75% quantile and 25% quantile as top border and the bottom border, respectively. Twenty haplotypes were simulated for a total of 10,000 rounds with ρ(=4N e Lr p) of 1000 at L = 167 kb. (PDF 5 KB) [file 12859_2014_6539_MOESM2_ESM.pdf]

C

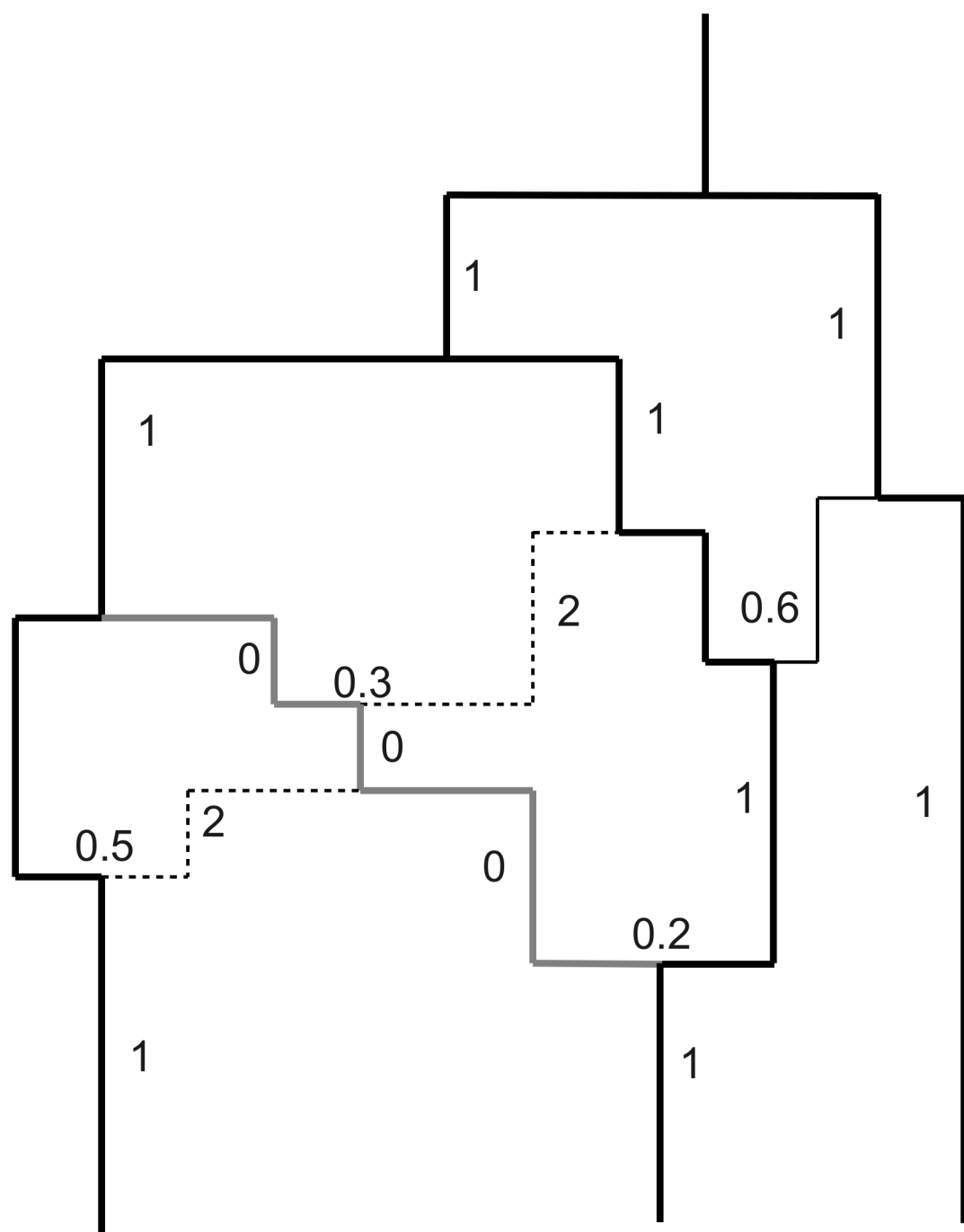

D

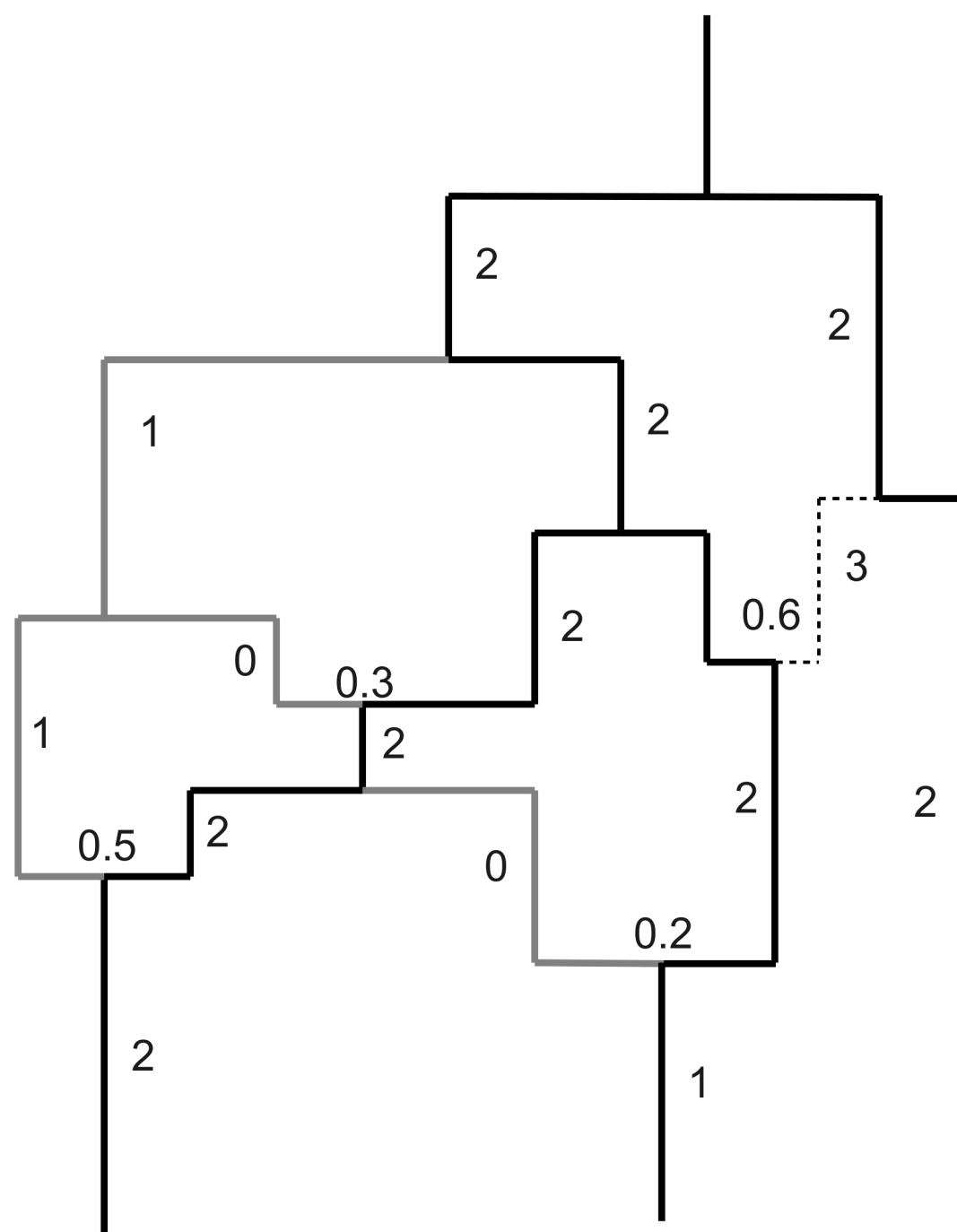

Supplement: Supplementary file 3 — Additional file 3: Figure S3: An example of SC method. An example of ARG. B,C and D describe the way of generating the ARG. The black thick branches make up the current tree. The gray branches are all old branches. The dashed lines are the path of the new branch. The black thin are un-simulated branches. The numbers in brackets display intervals which denote the ancestral materials carried by nearby branches. The numbers without brackets denote the recombination rates occur in the underlying nodes. In B,C, D, the numbers near edges are the labels of the SC method. (PDF 190 KB) [file 12859_2014_6539_MOESM3_ESM.pdf]

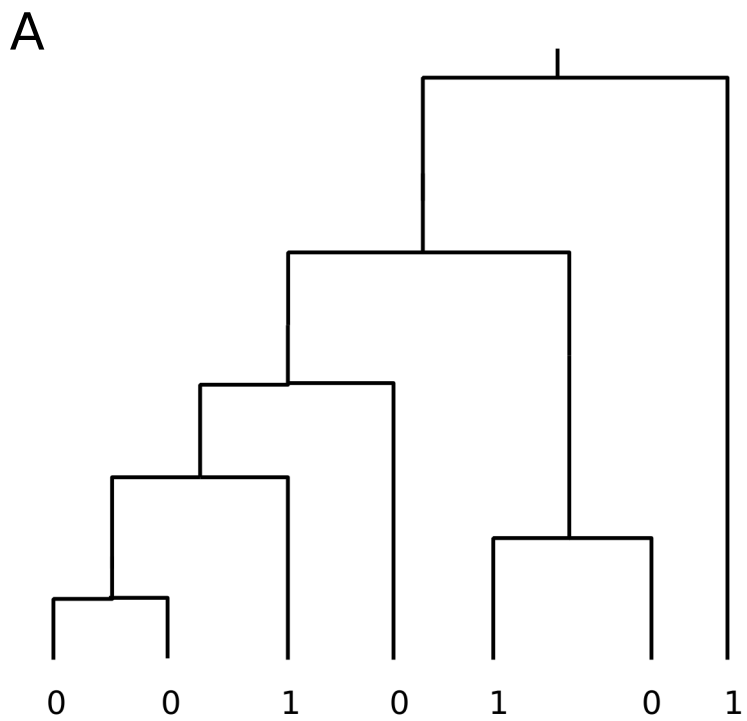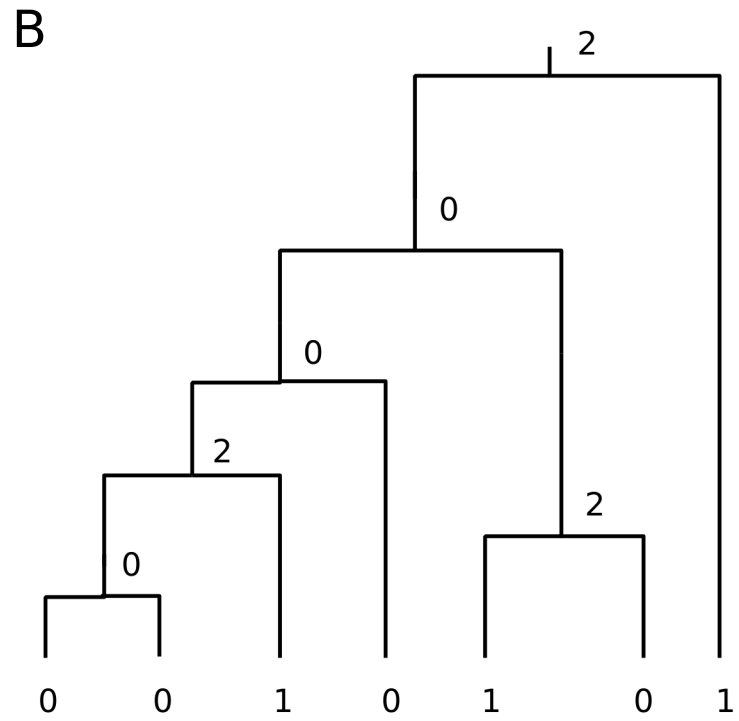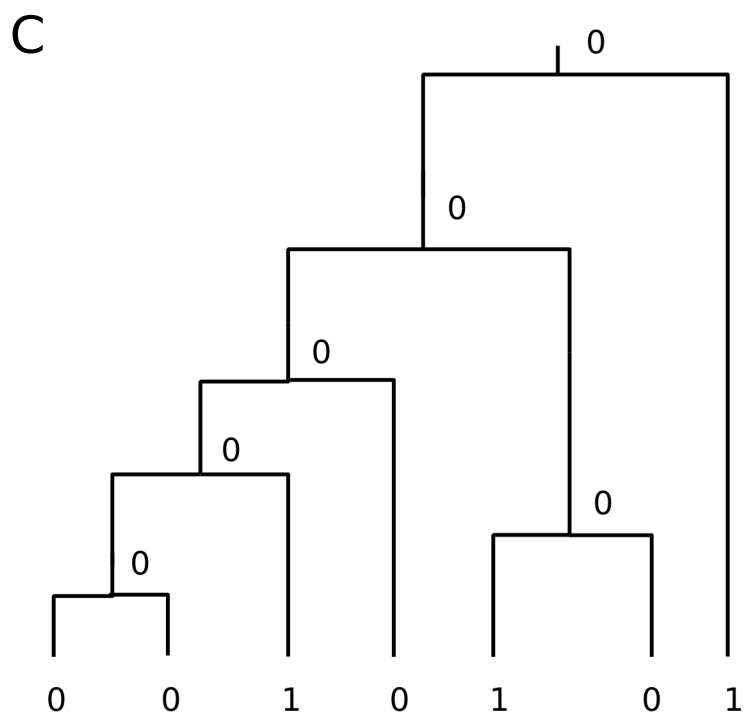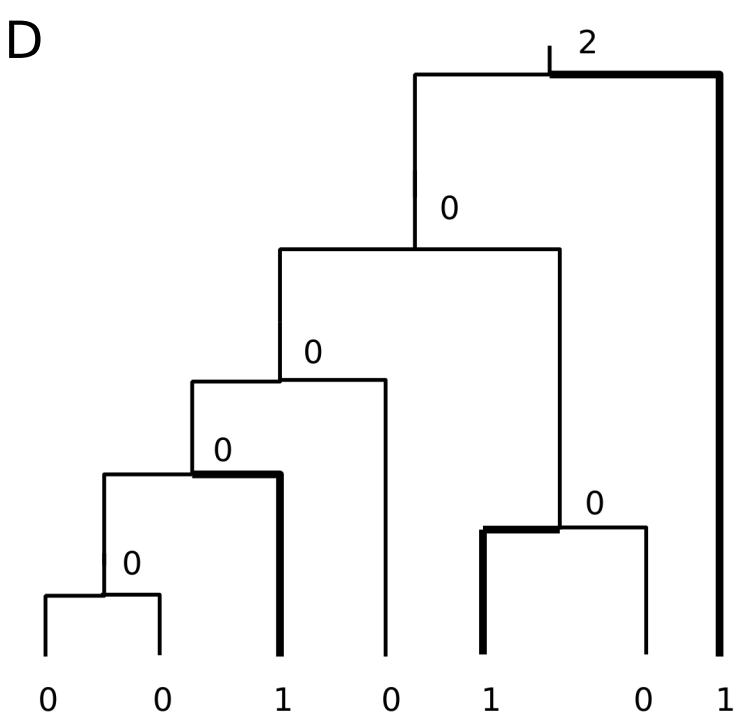

Supplement: Supplementary file 4 — Additional file 4: Figure S4: An example of the MMN algorithm. Step 1, value the leaf nodes with the sample. Step 2, value each node from bottom to top. Step 3, revalue each 2-valued node. Step 4, thick lines denotes mutation branches, get the MMN = 3. (PDF 54 KB) [file 12859_2014_6539_MOESM4_ESM.pdf]
